# Medical Sketches Taken in the Royal Navy

**Published:** 1812-06

**Authors:** James Robinson Scott

**Affiliations:** SURGEON, R. N.


*For the Medical and Physical Journal.*

### MEDICAL SKETCHES *taken in the* ROYAL NAVY, *by* JAMES ROBINSON SCOTT, SURGEON, R. N.

(Continued from p. 295.)

CONCERNING the propriety of using this evacuation (bleeding) in fever, there has been the greatest variety of opinions, and opposition of sentiment. More has been written, perhaps, on fever, than on any other disease. Theorists have produced many volumes, in their endeavors to discover its remote causes, to account for its phenomena. Hypotheses have been framed, which have had their day; these again have given place to others more plausible, but scarcely more satisfactory. The great father of medicine attended to *facts:* by simply minuting the various changes of disease, and observing what was most useful in removing them, he acted, perhaps, more wisely than many of our most eminent medical philosophers of even the present enlightened and scientific age; nevertheless, as I previously remarked, in the present day, that *theory* ought always to have its due force and weight, which is derived from a knowledge of *natural laws,* and may lead to a practice to be subsequently sanctioned by experience.

The great Dr. Cullen, whose practice was so extended, whose penetration was so acute, and whose medical erudition was so profound, by no means accounted either for the phenomena of fevers or those of inflammation, in his theories of these affections.

Brown treated fevers on *his* principles, with his copious and indiscriminate diffusible stimulus; simplicity was *his* forte, when he made a barometer of the human system. Darwin also had his particular hypothesis; the most rational, perhaps, of any in its application to the phenomena of fever.

To enter, however, into any disquisition on what writers have said on these subjects, would be improper in this place, and foreign to the design of this Sketch, which I intend merely as a hint to naval practitioners respecting the neglect of venesection, hoping that some among them, whose whose experience is more mature, and whose professional information is more extensive, might treat the subject in a minute and decided manner.

Without adverting to the endemics of tropical climates, I shall, in this concluding part of my view of depletion by the lancet, confine myself to a few observations on its utility in simple continued fever, attended by inflammatory action, such as is frequently met with aboard ship.

The treatment of this order of the Pyrexiæ requires, perhaps, more discrimination than that of the others. When a case of fever presents itself, *bleeding*, if from the habit of the patient it may be advisable, ought certainly to be used in the most prompt and speedy manner. I know practitioners so *completely prejudiced* against the lancet, that they, in *synochus*, in its incipient stage, though the patient were young and plethoric, would withhold this relief, "*from a dread of great debility being the result, and typhus supervening*." I must confess I am at a loss to account for the motives of such a line of practice, when it does occur; but I shall, by and by, give a case which affords a strong proof of its impropriety; a case where, conjoined with synochus, there was a strong determination to the head,—indeed such a state of the brain and vascular system as called loudly for venesection; yet this was delayed until the patient's life was in the most imminent danger. I knew one practitioner who administered wine and cordials to a patient in the incipient stage of inflammatory fever, accompanied with increased vascular action in the brain; pulse 130, and high delirium; while he bled the patient now and then, taking care not to take away much blood. This case was treated, after its removal into an hospital, with copious bleeding and abstinence, and was cured. The practitioner says, "I bled him; but, to guard against debility, I let him have his full allowance of grog and wine; and I gave him bark, tonics, and strengtheners." In another case, when Phrenitis had actually supervened, cardiac draughts were administered, and venesection withheld, though the pulse beat 150 in a minute, full and strong, with furious madness!

Case the 1st.—P. L. private, Royal Marines, aged about 25 years, rather athletic, and of a sanguineous temperament, had been stationed on-shore for some time, where he probably had been in the habit of living more freely than when aboard; however, on his again joining his ship, he applied in the sick bay for medical assistance, at the time of the surgeon's visiting the sick, at nine o'clock in the morning. His face was flushed, his skin hot, pulse 110, with some thirst; bowels rather constipated: he also complained of drowsiness, dull heavy pain in the head, but no rigors. A purgative was given, and, after its operation, he was to take occasionally a diaphoretic solution of tartrate of antimony. I expressed (on being consulted) that I thought he ought to be bled pretty freely; but this was objected to. Second day of his illness: Purgative has operated, skin hot, pulse 120, and rather wiry; thirst. Contr. In the evening no better, pain in the head, and face flushed. Third day, morning: Symptoms as yesterday; pulse still 120. ℞. Emplast. vesicat. mag. scrob. cord. applic. At noon appeared very like a person half intoxicated; thirst, heat, and frequent pulse, still continued. I again proposed VS. to the gentleman on whom his care properly devolved, but was told that "bleeding should not be used, unless in *urgent* cases, and then with great caution." I stated the quick pulse, lethargic state, or a tendency to it, with the pain in his head, &c. as indicative of meningeal inflammation, or pressure on the brain; but I was told that perhaps drinking was the cause, and to beware of being imposed on. The patient, I found, on inquiry, had not been out of the sick bay, since his first application, but once or twice, and then only for a few minutes; besides, he was a sober, quiet, man, one whom there was no cause to suspect of drunkenness; he had been peremptorily told not to taste wine or spirits. I ordered him to be kept in the sick bay constantly, and I saw him myself every two or three hours, and sometimes oftener, noting particularly the progress of his complaint. Third evening: no better. ℞. Pulv. antimonial gr. iv. h. s. s. Fourth day, morning: some diaphoresis; very drowsy and listless; pulse the same or nearly so, in frequency, but more full than at first; thirst; blister dressed. I again proposed VS. but it was rejected. Evening: Contr. med. diaphoretic. Fifth day: Thirst diminished; symptoms as before. Contr. med. Sixth morning: He was delirious during the night; perspires more freely. Rasura capitis, et emplast. vesicat. mag. toto capiti applicand. Constipation. A purgative ordered. Evening: Purgative operated; part of the day he has been comatose; stools and urine involuntary: no VS. Seventh: Through the night of the sixth day he has passed stools and urine involuntarily; does not regard any thing near him; eyes heavy; pulse 110. On my strongly pressing venesection, it was now agreed, that a few ounces might be taken away. Seeing that our patient's life was in danger, I determined to trifle no longer; I accordingly opened a vein, and took away as much blood as I thought the patient could bear to lose, considering all circumstances, which, when weighed, was 26 ounces avoirdupois. He did not faint under the operation, but his pulse sunk low; it soon afterwards rose again, though less strong. As I was well aware of the nature of his complaint, I concluded that the loss of 8 or even 12 ounces of blood, could be of little use; I wished to remove vascular distension and excessive stimulation; and this quantity of blood was sufficient palpably to affect the system, *as designed.*

As deliquium animi would here have been probably injurious, I gave the patient the following draught immediately after I bled him:

℞. Sp. Lavend. Comp. ℨſs.

Aq. Ammon. pur. ℈j.

— Menth. pip. ℥ſs. M.

I strictly enjoined abstinence from food; nothing for a day or two but barley-water; his blister was dressed, and he was put to bed. Evening: Contr. med. ut antea; pulse 110; he is very lethargic. Eighth day: Delirium slight through the night; skin cooler; thirst continues; pulse 90, and feeble. Evening: ditto, ditto. The surgeon was surprised at the quantity of blood taken away, and indeed alarmed. Ninth: Feels light and easy; speaks rationally, and wishes much for food. Tenth: Out of bed; light food, sparingly allowed.

I need not tire my readers with a continuance of these minutes; in another week the patient was discharged fit for his duty, and I was gratified by the reflection that I had probably assisted in saving his life. And here I beg leave to remark, that a medical man could scarcely have been placed in a situation more grating to his feelings than I was: there was not much discrimination required, surely, to know in this case what line of practice to pursue, it was glaringly obvious; yet venesection was postponed till it might have been too late, from fear of the patient's falling into *typhus.* The gentleman who superintended this man's treatment, saw him generally twice a-day, and never oftener than three times, and then only for half a minute or a minute; in fact, he could scarcely, at first, be persuaded that his illness was not feigned, until he became delirious. Being assistant surgeon of the ship, I could not insist, though it was my duty strongly to remonstrate. Reflecting that I had from neglect of this evacuation been at the point of death, and that its not being used in two cases would ultimately, in all probability, cost me my life, I thought I should have acted highly improperly had I passed this treatment over silently at the time. I think, let this case be viewed in every light, it still must strongly prove the danger, in such obvious inflammatory affections, of neglecting depletion by the lancet.

The dread of typhus has induced many practitioners, even in impending visceral inflammation, to neglect direct depletion; and others to stimulate, when they should very sparingly administer either food or other stimuli. It is by no means uncommon for a bigotted Brunonian, (I say a bigotted Brunonian, for that system owes its existence to sublime philosophical conception,) to give wine, or ardent spirits, when he should administer the dieta aquæa. This proceeds from the great chance of mistaking, in practice, the symptoms, kinds, and degrees of debility, treated of with so much apparent ease, and apparently so exceedingly simplified, in the Brunonian system.

In those cases of fever, of which men of robust constitutions are the subjects, occurring on-board ship, the exciting causes of which may be cold, or considerable exertion, after the system has been somewhat debilitated by unusually free living in harbor; venesection is generally found, if used in time, to be the best remedy: it mostly prevents the accession of some visceral inflammation, and the impropriety of its use I can scarcely think can be inferred from the sudden degree of debility existing, as this is the effect of the general inflammatory diathesis; for, admitting that a man accustomed to temperate living, or a certain regular application of stimulus, by encreasing that stimulus, brings on a degree of indirect debility in the moving fibres of his system; so that, on the application of the exciting cause, dangerous and excessive re-action takes place between the circulating powers and the circulated fluid; we are not to restore the stimulus which caused that indirect debility, we are to withhold it; nay, more, if we expect success, we must abstract a portion of the stimulus in the system, re-acting with such violence on the moving powers of that system.

When persons whose systems are debilitated, by the long and continued use of ardent spirits, or by the excessive use of it for a short period, are seized with inflammatory affections, *of which the use of ardent spirits is the most frequent and fatal predisposing cause;* the danger is most certainly greater in such persons, and the means of subduing it should be proportionably prompt; yet, on the accession of inflammation, a greater degree of debility will exist than in others, from the state of the nervous system. In dysentery, when the predisposition is caused by the use of ardent spirits, if great debility exists, (whether cold, or the actual contagion has excited the disease,) should we administer the patient's accustomed and poisonous beverage, for fear its sudden removal would injure him? Should we refrain from bleeding him, knowing the practice to be useful, merely because because a prejudiced lover of the diffusible stimulus might say, do not take away the stimulus, for fear of the sudden accumulation of excitability, &c.? Not reflecting that this stimulus has been one cause of the disease, and that the blood distending the vessels of the diseased parts is another; and that the system acquires a new and general stimulus—the accumulation of caloric in it.

Indeed we cannot be too careful to avoid, *after* inflammation has been successfully treated by venesection, the incautious exhibition of stimuli; increasing the food gradatim, for here we do produce fresh re-action, from the accumulated excitability; and, wherever inflammation has once occurred, the *vascular system* of that part *being debilitated by the former inflammation*, it will be very likely to occur again.

To illustrate the effect of mistaken notions of debility, in *actual practice*, I shall give a case, which many experienced men have thought a remarkable one, in which the patient nearly lost his life. The practitioner who superintended it, must have mistaken a genus of the class Pyrexiæ for one of the class Neuroses, for nothing else could have (in my humble opinion) prompted him to persevere in it.

Case 2d.—Mr. J. C. G. B——d, midshipman, aged 22 years, of a short gross make, and very full habit, short neck, and florid complexion, joined his majesty's ship the ———, lying in the river Tagus, in the beginning of February, 1811. He had been living on-shore for a considerable time before, and, being a young man addicted to every species of excess, particularly to drinking, there was every reason to believe he had injured his health when ashore. He first had slight fever with head-ach, for which he had a purgative administered, with injunctions to abstain from spirits in any form; however, he persisted in drinking occasionally, and was soon observed to wander very much in his conversation, and complained often of impaired vision, seeing double, &c. As I noticed a strong determination to the head, and was almost continually in his company, I was resolved to watch the progress of the complaint closely, though I foresaw that I should not have it in *my* power to benefit him much.

He was put on the sick list, and continued to walk about, and eat as heartily as usual, though he had occasional fits of vomiting; till, on the 9th day of February, when sitting at his tea, he fell back into my arms in a strong fit of epilepsy, but it was of short continuance; his face was of a purple hue during the fit, and from this moment his senses continued in a disordered state. On inquiry from some who had been ship-mates with him for a long time, I found that he had had not had epilepsy before; but that, "*from drinking, he had been deranged for some time in his head,*" about two years before. This fit was undoubtedly symptomatic, as the sequel will show; and, to prevent the accession of another, as well as to lessen cerebral irritation, his pulse being then 110, and full, and having been so for two or three days, I proposed, on minutely describing his case to the surgeon, copious direct depletion. I gave my opinion as founded on continued observation of the accession of the disease, and my knowledge of his mode of living. But, before I proceed in narrating this case, I ought to mention, that our patient had laboured under great dejection of mind for some time, owing chiefly to his not getting as much grog as he wanted, and also to regret, arising from domestic troubles. To this melancholy disposition the surgeon paid the chief attention; and, even after the state of his pulse was known to him, and the other symptoms, this opinion still prevailed, and governed his treatment of the case. Our patient, when first taken ill, had frightful dreams, sleepless nights, &c. and reflected much on his past improper conduct, so that I had my fears of his being inclined to commit suicide. VS. was not permitted, and I foresaw the result; I was consequently necessitated to be a spectator of the case, and, had it terminated fatally, I must have been placed in a most uneasy situation.

Feb. 10th. Bowels regular; pulse 110; face flushed; vision confused; incoherent in his discourse; nothing prescribed. I now have succeeded in preventing his getting any grog or wine, and I have enforced the antiphlogistic regimen.

11th. Much the same as yesterday; pulse still 110.

℞. T. Opii gtt. xxx. Vin. Ant. gtt. xl. h. h. s.

12th. Had another epileptic fit last night, and fell out of his hammock; worse to-day; will not go to bed; walks about, and speaks very wildly. Venesection proposed again by me, but not agreed to. Pulse 110, and just as before. Evening, a purgative prescribed.

13th. Pulse as before; skin very hot; face florid; continually talking incoherently, and fancies he sees ropes, pictures, machinery, &c. on the table, and endeavors to take them in his hands; appetite good, but not permitted to eat as he wishes. The surgeon ordered him a cardiac draught at bed-time. On our conversing together about the case, the surgeon thinks that he labours under "*nervous debility,*" and requires some stimulus occasionally, such as, T. Lavend. ℈j. Aq. Amm. pur. ʒss. Aq. ℥ij. ft. haut. h. s. s.

14th. Got out of bed this morning at three o'clock, dressed himself himself in his full dress, and put on his side-arms, insisting that he was to dine with the captain, and was quite outrageous on the sentry striving to put him in bed. I rose up, and by flattery and threats got him pacified. At noon, has no trace of recollection as to the ship, his messmates, &c. and does not know their names; is rather more quiet than in the morning. Emplast. vesic. mag. capiti toto applicand. While there is yet any prospect of being able to bleed him, I have again adverted to its propriety, but it is thought inadmissible. Evening, quite outrageous; we are obliged to use the strait waistcoat, and it has taken five strong persons to put it on; there is great muscular exertion, grinding his teeth, eyes suffused, face red, pulse actually 150; in fact, there are now unequivocal existing symptoms of Phrenitis: his pulse is strong and full.

15th. He has been restless all night, constantly struggling to get out of his cot, so that it was necessary for two sentries to be placed over him, though he was tied in the cot, by means of flannel bandages, which I passed round his wrists, ancles, and waist, and secured to the head and foot of the cot; nevertheless, he had them all loose in the morning; his blister has risen well; he is very thirsty, and his urine and stools are involuntary. I have again proposed bleeding, and that in the art. temporales, which I could still have accomplished, as I have got him to recognise me a little, by being somewhat severe, such as by throwing cold water on his face, &c. Bleeding rejected again by the surgeon. I now resolved to propose VS. no more.

16th. During last night stools and urine involuntary; pulse 150, and full; face exceedingly red, and apparently inflamed on the surface; blister dressed. He has been exceedingly furious last night; I was obliged to get out of bed at twelve o'clock to strive to pacify him; he struck the men who were attending him, used a vast portion of muscular strength, more indeed than I thought he could have possessed, and raved incessantly. His face exhibits an erysipelatous appearance, and he has begun to perspire pretty freely; pulse as usual.

17th. Muscular exertion still continues, but is not so great as the preceding night; urine and stools still involuntary, but he has perspired most profusely last night, his shirt and bed-clothes being wet through, though his linen was twice changed; he has also diarrhœa.

18th. On going to him this morning, I found his pulse only 75, extended on his back, and unable to speak, and a cold sweat over him. I thought his disease had terminated fatally. I was however disappointed most agreeably; for, the the diarrhœa and perspiration supervening, together with the erysipelatous affection of his face, happened in his case fortunately to be favorable terminations of the excessive vascular action; the translation of the inflammation to his face, in some degree, was highly favorable, and nature was relieved by the excessive perspiration and diarrhœa from the plethora and irritation, which the lancet, if timely used, would, in all probability, have removed.

19th. He has continued extremely weak since yesterday, scarcely able to speak; pulse 75; skin cool, and has recovered his recollection; says he forgets every thing since December, and only remembers living ashore at that time. This is a most extraordinary fact, for, to my certain knowledge, he was for ten or twelve days before he had the epileptic fit apparently without any affection of the brain, or any derangement of his perceptive faculties. This either proves, that all along from his coming on-board in January, and even before that time, he had been laboring under cerebral irritation; or that his illness had obliterated all recollection of what happened to him since he came on-board, which was about the 12th of January. Our patient was strictly enjoined rest, and a continuance of the antiphlogistic regimen: this, with an occasional laxative, was all the treatment which was found necessary, until he gradually recovered strength, which was in about three weeks: he was well enough to go to his duty in five weeks from the accession of his illness.

This case, although it did not terminate fatally, shews the imminent danger of leaving such cases to nature, or of stimulating when we should deplete. The symptoms were truly alarming, and, owing probably to some peculiarity of constitution, nature was enabled to arrest the further progress of the disease; and to this the patient is, I think, indebted for his recovery. The gentleman who superintended the case, first prescribed cardiac draughts; and, finding them useless, he persisted in leaving the case to Nature. But, if he mistook this case for one of the genera of the order vesaniæ, class neuroses, why did he use a blister for the head? In fact, he *did* consider the case as such. His words were nearly to this effect: "We must not debilitate too much; the young man's mind is affected; he has been reflecting on his conduct, &c. and his nerves are weak; besides he has, I hear, been deranged *in his mind* before."—So he had been, for intemperance had produced excessive vascular action in the brain, or its meninges. I have detailed this case, as well as the preceding, to shew that where VS. is most

 palpably palpably indicated, (and who can deny that it was not in both these cases?) it is frequently, from rooted prejudice alone, withheld until the patient is placed upon the very brink of destruction, and sometimes until he actually falls down its precipice. The subject of the last case, I am grieved to state, soon reverted to his former habit of using the diffusible stimulus, though impressively warned of the future fate which probably awaited him. His disease is most likely to return again with redoubled violence.

I have now stated my opinions relative to the danger of neglecting, in acute diseases, an important evacuation—the anchor of hope in such diseases. I have animadverted on the neglect of it as occurring under my own observation in the navy, where the subjects of such diseases are so exceedingly liable to their most violent attacks, and where, from their habit robust and healthy (generally speaking), they require that, when alarming symptoms present themselves, this evacuation, if indispensable, must be pretty copious; a neglect censured as it is, and ought to be, by men of the most extensive practice and discriminating faculties in the navy; and lastly, to a neglect which has done my own constitution irremediable injury, not merely in the instance alluded to in No. 157 of this Journal, but in another severe attack which I had in the winter of 1808–9, which terminated in expectoration, adhesions, alarming and at that time apparently incurable debility: I had night sweats and hectic, and removal to a milder climate alone saved my life. Not being aware of my danger at that time; not having given the subject that ample consideration which I since have; I suffered myself to be persuaded into a false security. Nature was left to herself, and the consequences have been to me truly deplorable: it is a full twelve-month since I have ceased to be free, even for a day, from the most painful and distressing symptoms attendant on thoracic inflammation, repeated often; which state of the lungs I now labor under (termed by Mr. Abernethy *infarction*), and which I much doubt can be ever removed or arrested in its progress, by any plan of treatment whatever.

No man situated as I then was, on a sick bed, can properly be his own physician; the resolution wavers, the judgment is clouded, the mind is agitated. I trusted to one person; confident in himself, he neither called in another, nor advised me to do so, neither did he think mine, at that time, a *pressing hospital case*. The sequel shewed me my error when too late.

My situation in life, it is true, exposed me to the exciting causes of thoracic inflammation; but every man's situation exposes him to some cause of some disease. I was always from constitution predisposed to these affections, but in two instances the most palpable injury was done me by not removing the proximate cause, as far as medical practice sanctioned the means which were neglected. Of little use is our scholastic exercise, our instructions in medicine, our disquisitions, our attachment to one system or the other, if we cannot apply the most rational of these in practice, so as to benefit our fellow men. I have spoken of the Brunonian system. I know that it has a number of enthusiastic followers, and I also know facts, the result of erroneous application of its principles, which prove to me that it is perhaps one of the most dangerous systems that was ever produced to the world. I say dangerous, because improperly acted on by its implicit believers; though the system itself is the production of a sublime Philosopher, and, with various modifications, continues at present the foundation of the reasonings and opinions of some of the most eminent pathologists. I conversed with a practitioner, to whom I said "that Brown's attempt at simplification in medical science, rendered his system in its application to practice dangerous, if not absurd;" but he liked it for its simplicity, he said, more than for any thing else. Indeed the classifications of disease by nosologists are liable to one great objection, that they are of no practical use, that they do not in any case sufficiently define diseases, so as by giving the peculiar marks of any particular deviation from health, to correctly distinguish it from every other. In fact, from the innumerable varieties in the human constitution, influenced by as innumerable causes, and subject to such changes from the endless modifications of disease; and from our ignorance of so many of the laws by which the animal machine is governed, we cannot at present give what may be justly called a *definition* of *any disease*.

Nosology is useful by assisting the memory, and by saving trouble and confusion in medical intercourse; but no system of nosology can be correct which describes and classes diseases according to their apparent or imaginary qualities: it must be unphilosophical at the least. Thus, although we know what is meant by phthisis, the title is as unphilosophical as is the classification of that disease in the order hæmorrhagiæ of the class pyrexiæ. The constitution of one man must always differ from another, really though not perhaps apparently, in some degree; disease will always have some shade of difference, just as the system, acted on by by it, differs in general or partial structure, i. e. in aboriginal excitability; and it is by minutely observing particular circumstances, and by industriously dwelling on facts, that a practitioner only can confer benefit on mankind, and fulfil the ends of his profession.

In the course of this essay I adverted to VS. in thoracic inflammation, rheumatism, dysentery, and synochus. I may have omitted various minutiæ, not because I was unaware of them, but that I thought them of minor consideration, as they regarded the question of copious direct depletion generally, or particularly in the diseases which I have mentioned.

I have given one case where the quantity of blood taken away at the first bleeding was very great. Perhaps I might never meet again with so very athletic and sanguineous a subject, attacked so violently as he was, his pulse being, as I before stated, exceedingly full, strong, and rapid. This patient, nevertheless, did not faint while I was bleeding him, but a short time after the operation; his pulse getting slow towards the end of the operation. I considered, that, with such a subject, the ordinary evacuation of an adult in such an affection would scarcely have the desired effect; in proportion to the vigor of this man's habit, and the severity of his pain in inspiration, the danger to be dreaded was great. And did not his subsequent, almost immediate, recovery prove that the treatment was such as the case required?

I must beg leave, before I conclude, to observe, that for the last two years the subject of this essay has employed my most serious reflection. I have sought for information, practical and theoretical; I have given my own opinions candidly; I have related only a few of the facts which attracted my observation, and employed my reflection; nevertheless I consider them enough to illustrate my positions, and sanction my conclusions. If the facts related, and particularly the statement of my own sufferings, do not excuse me in publishing it, nothing else can.

I did not make any allusion to the use of blisters in these formidable affections. I consider them as matters of course, particularly in pneumonia, where they are often of infinite service.

With respect to the state of the pulse, I shall make a few observations, in the affections spoken of, as indicative of the strong propriety of using direct depletion.

From what I have observed since I turned my attention particularly to pneumonia, and from what I have advanced in this sketch, added to the generally received opinions respecting these diseases, I shall draw these conclusions,

1st. That pain in inspiration, laborious respiration accompanied with a sense of load in the chest, and a pulse more rapid than natural, together with increased heat of surface, are sufficient, if they appear where there is no chronic exhaustion present, to warrant the timely and free use of the lancet. Pain in the breast, increased heat of surface, and quick circulation, are then the principal indications of existing thoracic inflammation:

2d. That the pulse may be, in these diseases, rapid, small, and hard; rapid, strong, and full; rapid, small, and weak; and quick, full, and soft. The first kind of pulse, rapid, small, and hard, may occur in incipient thoracic inflammation, and is strongly indicative of excessive re-action in the arterial system. This pulse will occur most frequently in subjects of a spare habit, young, irritable, and sanguineous withal. Bleeding will often convert this pulse into a stronger, fuller, but slower, pulse. The second pulse is also indicative of powerful stimulation of the vascular system; and will be present in the strong athletic habit, unhurt by original disease. This will as strongly indicate VS. in the first stage. The quick weak pulse may also indicate VS. but this pulse is likely to characterise debility, or a state of chronic exhaustion, suffering under the additional stimulus of the inflammation. Here VS. must be used with great caution, and the other auxiliaries called in; yet this pulse will rise if VS. prove useful, i. e. it will become stronger and slower.

3d. The pulse is not the criterion which should solely, or even chiefly, direct the plan of treatment. If no previous severe exhaustion has taken place, a pulse merely rapid, if conjoined with heat of skin, difficulty of breathing, and pain in any part of the cavity of the thorax, warrant the prompt application of direct depletion.

I perhaps have stated, that I approved of taking away a quantity of blood larger than many practitioners would sanction. From whatever observations I may have made, and from a consideration of the properties of the blood, the different states of the living organized system, and their actions on each other, I was induced to form an opinion that a large quantity of blood taken away in the incipient stage of an acute disease,* is productive of more real good, and causes less permanent debility, than small evacuations, calculated to do little good, and finally to cause the loss of more blood to the patient, than the other method. I have every reason to believe that more lives might be saved, and that without subsequent disease, by this practice.

* At one or two bleedings.

For

For the first bleeding, it is a delicate adult, from whom, in thoracic inflammation, it will not be necessary to abstract sixteen ounces; and perhaps from the most athletic and sanguineous thirty ounces, or even two pounds. In general, from an adult of moderate size and strength, if not previously exhausted by chronic disease, I have taken twenty-four ounces; and this quantity I never found permanently to affect the patient's strength, but often to arrest the progress of the inflammation, and to prevent subsequent depletions. I had, the other day, a patient subject to drinking, who was attacked with pneumonia in a manner rather alarming, and with great sudden debility. I took away twenty-four ounces of blood in the very commencement, enjoined low diet and diluents, and, without giving him any medicines whatever, perspiration supervened, his pain left him, and in seven days he was fit for duty. He was a man of but moderate strength. In a very athletic subject I should have taken, without hesitation, thirty ounces away.

To conclude, if we have the best reasons for using a certain practice, when our patient is in danger, let us not procrastinate: the watch-word in medical practice ought to be—promptitude! By neglect in one instance, nature may be left to toil for years of misery, and at last sink into death; the frame reduced to a shadow, and the organs of life altered by disease and exhausted by ineffectual struggles. "Whatsoever thy hand findeth to do, do it with thy might," is an injunction not to be neglected by the physician above all other men:—

"We are such stuff as dreams are made of,
"And our little life is rounded with a sleep."

